# Two clusters of residues contribute to the activity and substrate specificity of Fm1, a bifunctional oleate and linoleate desaturase of fungal origin

**DOI:** 10.1074/jbc.RA118.005972

**Published:** 2018-10-22

**Authors:** Yuanheng Cai, Xiao-Hong Yu, Qun Liu, Chang-Jun Liu, John Shanklin

**Affiliations:** From the ‡ Biochemistry and Cell Biology Department, Stony Brook University, Stony Brook, New York 11794 and; § Biology Department, Brookhaven National Laboratory, Upton, New York 11973

**Keywords:** structure-function, substrate specificity, fatty acid metabolism, dehydrogenase, lipid metabolism, Δ12 and ω3 desaturases, Fm1, polyunsaturated fatty acid, structural determinants, substrate specificity, phospholipid, regioselectivity, bifunctional desaturase

## Abstract

Polyunsaturated fatty acids (PUFAs) have important industrial, physiological, and nutritional properties. Plants use the sequential activities of FAD2 and FAD3 desaturases to convert 18:1Δ9 to the important PUFA 18:3Δ9,12,15, whereas the fungus *Fusarium verticillioides* 7600 uses the bifunctional desaturase Fm1 for both reactions. Here, we used a combination of sequence comparisons, structural modeling, and mutagenesis experiments to investigate Fm1's regioselectivity and identified two functionally relevant clusters of residues that contribute to Fm1 activity. We found that cluster I (Leu^153^, Phe^157^, and His^194^), located near the catalytic iron ions, predominantly affects activity, whereas cluster II (Tyr^280^, His^284^, and Leu^287^), located in a helix forming the entrance to the substrate-binding pocket, mainly specifies 15-desaturation. Individual or combined substitutions of cluster II residues substantially reduced 15-desaturation. The combination of F157W from cluster I with Y280L, H284V, and L287T from cluster II created an increased-activity variant that almost completely lost the ability to desaturate at C15 and acted almost exclusively as a 12-desaturase. No variants were identified in which 15-desaturation occurred in the absence of 12-desaturation. Fm1 displayed only traces of activity with C16 substrate, but several cluster I variants exhibited increased activity with both 18:1 and 16:1 substrates, converting 16:1Δ9 to 16:3Δ9,12,15, consistent with Fm1 performing sequential *v* + 3 desaturation reactions at C12 and then C15. We propose that cluster II residues interact with the substrate headgroup when the acyl chain contains both Δ9 and Δ12 double bonds, in which case C15 becomes positioned adjacent to the di-iron site enabling a second *v* + 3 desaturation.

## Introduction

Polyunsaturated fatty acids (PUFAs)^2^ are a class of fatty acids that contain two or more double bonds in their fatty acyl chains that have important industrial, physiological, and nutritional properties. For example, very long chain (VLC-) PUFAs are involved in the regulation of human brain development (1, 2) as well as other cellular functions such as biosynthesis of cellular membrane phospholipids (3), modulating cell signaling (4), and serving as signaling messengers (5). Linoleic acids (LA) and α-linolenic acids (ALA) are the precursors of physiologically important VLC-PUFAs that are produced by various reactions, including desaturation, chain elongation (6), and β-oxidation (7), in mammals. They are deemed as essential fatty acids because mammals lack the desaturases for producing them, and they must be obtained from dietary sources (8). LA and ALA are also industrially important drying agents that are widely used in paints and varnishes (9). In nature, LA can be synthesized by introducing a *cis*-double bond at Δ12 position in oleic acids. Further insertion of a double bond at Δ15(ω3)-position in LA gives rise to ALA. The main dietary source for obtaining VLC-PUFAs is marine fish. However, with increasing demand for fish oil, other sustainable sources of VLC-PUFAs are needed (10). Oilseeds and/or microorganisms represent appealing alternatives to accumulating VLC-PUFAs.

In plants, the majority of PUFAs are synthesized by desaturases in the endoplasmic reticulum (11), *i.e.* the oleate desaturase FAD2 and the linoleate desaturase FAD3. These two endoplasmic reticulum membrane–bound fatty acid desaturases (12) contain a di-iron cluster at their catalytic centers. The metal ions are coordinated by three histidine-containing motifs (13). For desaturation, an electron transport system composed of cytochrome *b*_5_ and NADH-dependent cytochrome *b*_5_ reductase provides electrons (14–18), and the iron atoms and a molecular oxygen form an activated complex that abstracts hydrogens from two adjacent methylene groups and inserts a double bond in the fatty acyl chain (13). Both FAD2 and FAD3 are methyl-end desaturases, which insert a double bond between an existing double bond and the methyl end (19). Covello and co-workers (20, 21) classified fatty acid desaturases according to their desaturation positions: a Δ*x* desaturase inserts a double bond *x* carbons from the carboxyl end; an ω*x*-desaturase puts a double bond *x* carbons from the methyl end; and a *v* ± *x* desaturase uses an existing double bond as a reference, desaturating *x* carbons toward the methyl end relative to the existing double bond (*v* + *x*) or the carboxyl end (*v* − *x*). FAD2 inserts a double bond three carbons toward the methyl end from an existing double bond, in most cases a Δ9 double bond; therefore, it is considered as a *v* + 3 desaturase (or a Δ12-desaturase in the case of 16:1Δ9 and 18:1Δ9), whereas FAD3 performs ω3-desaturation, which inserts a double bond three carbons from the methyl end, hence making it an ω3-desaturase. The membrane desaturases share highly similar overall structures and secondary structures comprising four transmembrane domains and a soluble catalytic domain; however, their functions can vary greatly. In particular, high regioselectivity is one of the most remarkable properties for fatty acid desaturases (22). It is of great interest to determine how these enzymes discriminate their substrates and perform regiospecific oxidation to lay the groundwork for engineering enzymes with novel desired functions.

Although FAD2 and FAD3 share the same overall structural motifs, they share only 40% sequence identity and 55% similarity at the amino acid level. Other membrane fatty acid desaturases from microbes have been reported to possess both Δ12- and Δ15(ω3)-desaturation activity; for instance, Damude *et al.* (2) described a bifunctional Δ12/ω3-desaturase from *Fusarium moniliforme* (subsequently renamed *Fusarium verticillioides* 7600) (Fm1), heterologous expression of which can produce substantial quantities of ALA in both *Yarrowia lipolytica* and soybean. These bifunctional enzymes provide an opportunity to investigate the structural/sequence determinants for their substrate specificity. For example, Hoffmann *et al.* (23) identified two consecutive domains that are either close to or participate in forming the active site of the *Aspergillus nidulans* bifunctional oleoyl-Δ12/linoleoyl-Δ15(ω3)-desaturase with respect to the regioselectivity. However, the detailed structural factors or amino acid determinants for regioselectivity as well as the mechanism of regioselectivity remain unresolved. Here, we report the identification of two clusters of residues in Fm1. Cluster I residues are close to the diiron center and principally affect the rate of desaturation, whereas cluster II residues are located at the opening of the substrate-binding pocket and likely interact with the substrate headgroup, establishing the substrate specificity of the enzymes. Alteration as few as two amino acids in the sequence of Fm1 can efficiently block the desaturation at the 15-position while retaining the 12-desaturation capability of the enzyme. Using a mutant with higher overall activity enabled the C16 desaturation products to be evaluated. 16:3 derived from such reactions have double bonds at the 9-, 12-, and 15-positions, showing that Fm1 performs two sequential *v* + 3 desaturation reactions.

## Results

### Identification of candidate residues within Fm1 responsible for the introduction of 12- and 15-double bonds

**Figure 1.**
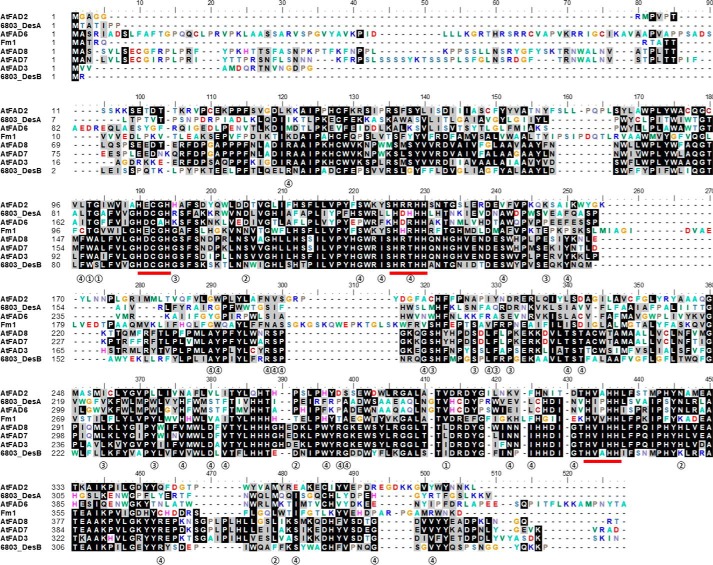
**Alignment of Δ12 and ω3 desaturation enzymes.** The three histidine boxes that are proposed to coordinate catalytic iron ions are *underlined red*. The sites that are selected for mutagenesis are categorized into four groups according to their consensus and numbered as groups ➀–➃ as shown in Fig. 2*A*. AtFAD2, 6803_DesA, and AtFAD6 are the Δ12-desaturases. AtFAD8, AtFAD7, AtFAD3, and 6803_DesB are the ω3-desaturases. Fm1 is the bifunctional Δ12/ω3-desaturase.

**Figure 2.**
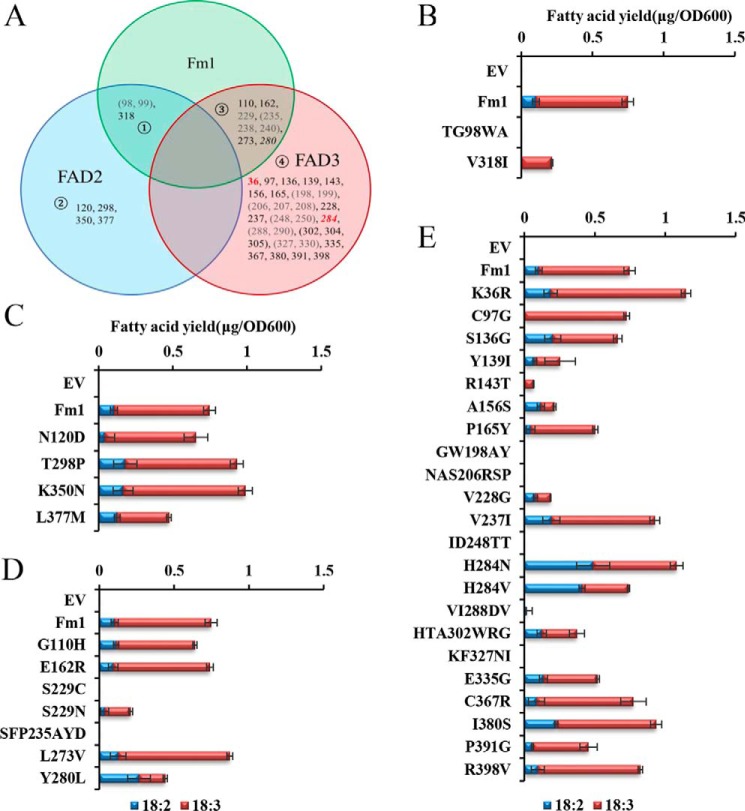
**Analysis of conserved residues that contribute to desaturation specificity.** *A*, Venn diagram of conserved residues that are shared by different groups of desaturases (➀–➃). Δ12-Desaturases are represented by FAD2, and ω3-desaturases are represented by FAD3. The positions in *parentheses* are combined and investigated as a single mutation as they are very close in sequence. *Stacked bars* represent activities of mutants for residue conservation between Fm1 and Δ12-desaturases (*B*), Δ12-desaturases only (*C*), Fm1 and ω3-desaturases (*D*), and ω3-desaturases only (*E*). The activities are presented as fatty acid yields (μg of fatty acids produced per *A*_600_ of cells). *Error bars* show the S.D. from three independent replicates. Mutations with greater than 125% of Fm1 activity are labeled *red* in *A*, whereas those that result in complete loss of Fm1 activity are labeled *gray. Italic* positions show the mutations that change product ALA/LA ratios relative to Fm1. *EV*, empty vector.

The amino acid sequences for membrane-bound fatty acid desaturases and related enzymes share relatively low homology. This reflects their diversity with respect to selectivity toward headgroups, substrate chain lengths, regioselectivity, and functional outcome. Nonetheless, they all share a core conserved tripartite histidine motif that ligates the catalytic iron ions (13). Multiple sequence alignment reveals that the iron-coordinating histidine residues align well, whereas other portions of the sequences vary greatly (Fig. 1). The conserved sequence regions of membrane desaturases are likely related to the overall architecture of the desaturases, whereas the less-well conserved regions may contribute to their observed substrate specificities. We included 12- and 15(ω3)-desaturase sequences from *Arabidopsis* and *Synechocystis* together with Fm1 in the alignment to encompass the diversity of available sequences. These sequences can be divided into two groups: one with 12-desaturation activity, including AtFAD2, 6803_DesA, and AtFAD6, and the other with 15(ω3)-desaturation activity, containing AtFAD8, AtFAD7, AtFAD3, and 6803_DesB. Fm1 can be classified into both groups as it is capable of performing both 12- and 15(ω3)-desaturation with respect to its 18-carbon substrate; therefore, we reasoned that it could possess features in common with both groups, whereas the features that are not shared by the two groups could potentially confer Fm1 with bifunctionality. At any specific amino acid location, if the residue within one group is conserved but differs from that in the other group, the residue of Fm1 at this position was considered to be potentially functionally relevant. Our first round of analyses revealed 44 such positions (Fig. 2*A*). Specifically, the residues at Fm1 positions 36, 97, 110, 136, 139, 143, 156, 162, 165, 198, 199, 206, 207, 208, 228, 229, 235, 237, 238, 240, 248, 250, 273, 280, 284, 288, 290, 302, 304, 305, 327, 330, 335, 367, 380, 391, and 398 are common to 15(ω3)-desaturases (Fig. 2*A*, ➃), whereas positions 98, 99, 120, 298, 350, and 377 are conserved in 12-desaturases (Fig. 2*A*, ➁). Fm1 shares conservation with 15(ω3)-desaturases at positions 110, 162, 229, 235, 238, 240, 273, and 280 (Fig. 2*A*, ➂) and with 12-desaturases at positions 98, 99, and 318 (Fig. 2*A*, ➀), which could potentially contribute to determining desaturation selectivity.

### Site-directed mutagenesis

The above analyses reveal 44 sites that potentially contribute to the desaturation specificity/activity of Fm1. We therefore preformed site-directed mutagenesis to analyze each site's potential contribution to enzyme activity. At each site, if one group was conserved but differed from that in Fm1, the site in Fm1 was mutated to the residue equivalent to that of the consensus group. Alternatively, if the consensus was shared with Fm1, then the site in Fm1 was mutated to the equivalent residue from the second group (*i.e.* FAD2 for the 12-desaturase group and FAD3 for the 15-desaturase group). Because some sites are closely located in sequence, we simplified our analysis by treating them as single sites. Although mutations of the sites that are conserved between FAD2 (12-desaturation) enzymes and Fm1 led to dramatically impaired Fm1 activity (Fig. 2*B*), mutations at sites that are only conserved in FAD2 enzymes did not result in substantial changes (Fig. 2*C*). Interestingly, mutations at sites that are conserved in both Fm1 and 15(ω3)-desaturation enzymes or FAD3 enzymes had broad effects on Fm1 specificity (Fig. 2, *D* and *E*). Notably, mutations at positions 280 and 284 resulted in an altered product profile: Fm1 mainly produces ALA with an ALA/LA ratio of ∼0.86 (as calculated by the observed ALA/(ALA + LA) because all the ALA resulted from a second desaturation of LA), whereas mutations at both sites preferentially produce LA, reducing the ratio of ALA/LA. Particularly, Y280L showed significant reduction of the accumulation of ALA with an ALA/LA ratio of ∼0.4, although overall enzyme activity was compromised. However, although H284N altered Fm1's ratio of ALA/LA to 0.55, it resulted in an increase in overall activity by more than 30%. These results show that these residues can influence both activity and specificity, and their separation by only four amino acids suggests that they are likely located within the same secondary structural element.

### Homology modeling

**Figure 3.**
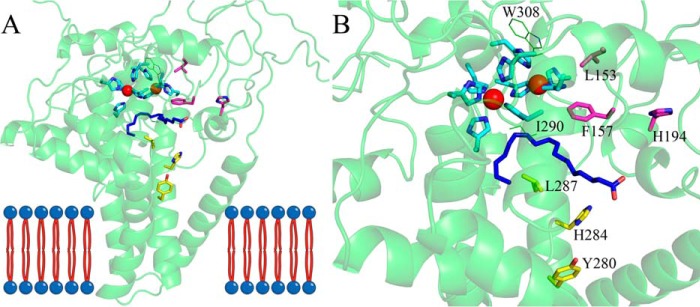
**Overall modeled structure of Fm1 (*A*) based on mouse stearoyl-CoA desaturase 1 crystal structure (PDB code 4YMK) and a close-up view of the catalytic domain (*B*).** The catalytic iron ions are shown as *red spheres*. The iron-coordinating histidine residues are shown as *cyan sticks*. A linoleoyl acyl chain shown in *blue sticks* was modeled into the substrate-binding pocket to schematically illustrate the binding of the substrate. Cluster I residues are shown as *magenta sticks*, and cluster II residues are shown as *yellow sticks*. Residues Ile^290^ and Trp^308^ are shown as *green lines*.

To investigate the possible spatial distribution of the sites described above, we modeled the three-dimensional structure of Fm1. The only membrane-bound fatty acid desaturase structures available to date are mammalian fatty acyl-CoA Δ9-desaturases (24, 25). In contrast to 12- and 15(ω3)-desaturases, these enzymes introduce the first double bond into fatty acyl chain at the Δ9-position and share very little sequence homology with Fm1 (∼18% identity and 30% similarity). However, the residues comprising their active sites are conserved, particularly the histidine residues that coordinate the catalytically essential iron ions. We therefore used the mouse fatty acyl-CoA Δ9-desaturase (mSCD1) structure as the template to model Fm1 3D structure and mapped the residues potentially related to Fm1 specificity/activity. As shown in Fig. 3*A*, the overall Fm1 model adopts a mushroom-like shape with four transmembrane helices and a cytosolic portion that possesses the substrate-binding channel and active site. Like the mammalian fatty acyl-CoA Δ9-desaturases, the substrate-binding channel of Fm1 is boomerang-shaped and can accommodate a *cis*-oleoyl or a *cis*-linoleoyl chain (Fig. 3*B*). The spatial distribution of nine histidine residues that coordinate two iron ions is also strictly conserved. In primary sequence, these nine residues are dispersed in four clusters, including motifs ^105^H*XXX*H^109^, ^141^H*XX*HH^145^, and ^337^H*XX*HH^341^ and a separate His^295^. The two iron ions are located adjacent to the bend in the substrate-binding pocket where they can activate a molecular oxygen to create the high-valence iron–oxygen adduct necessary to abstract two pro-*R* hydrogens to form the *cis*-double bond.

Interestingly, the model predicts that the two residues that affected the product profile of Fm1, *i.e.* 280 and 284, are in consecutive turns of the same helix (Fig. 3*B*). Moreover, this helix forms the entrance of the substrate-binding pocket, consistent with a potential role in substrate recognition and orientation.

### Mutagenesis based on structural analysis

**Figure 4.**
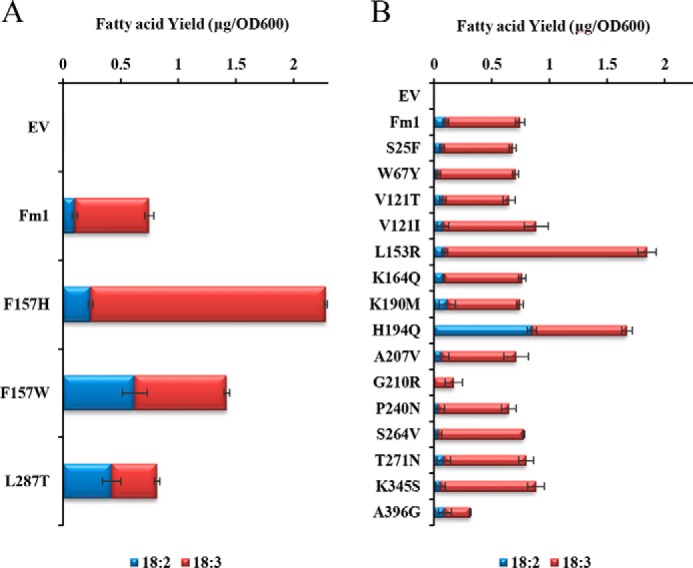
**Activity of mutants generated based on structural analyses (*A*) and comparison between Fm1 and Fm2 (*B*).** The activities are shown as mass of fatty acids produced per *A*_600_ of cells. *Error bars* show the S.D. from three independent replicates. *EV*, empty vector.

We hypothesized that the residues close to the iron ions could potentially fine-tune catalytic efficiency. We therefore analyzed residues within 5 Å of the iron ions in our model. Besides the conserved histidine residues, three residues appeared in our list, *i.e.* Phe^157^, Ile^290^, and Trp^308^ (Fig. 3*B*). Ile^290^ had already been investigated in the analysis of 15(ω3)-desaturase conserved sites in which mutation of Ile^290^ resulted in impaired Fm1 activity. Trp^308^ is strictly conserved in all the desaturases used for the alignment, and location 157 showed some diversity: for example, 6803_DesA, AtFAD6, and all the FAD3-like enzymes have a Trp at this position, whereas mSCD1 possesses an His. We tested the effects of the Phe^157^ mutations by substituting it with either Trp or His. Interestingly, both mutations significantly increase Fm1 activity (Fig. 4*A*). Specifically, substitution of His for Phe at position 157 increased Fm1 activity by ∼150%, and F157W increased activity by 65%. Moreover, F157W altered Fm1's product profile, producing ALA/LA at a ratio of 0.56, confirming that residues close to the catalytic iron ions can play an important role in both activity and specificity.

The residues in the helix that forms the entrance of the substrate-binding pocket seem to be critical for determining the product profile of Fm1 as revealed by Y280L and H284N/V. These two residues face the same direction of the helix. We hypothesized that other residues in the same helix facing the same direction could also affect the enzyme specificity in a similar way. We tested the effects of mutagenesis of Leu^287^, which is one turn away from His^284^. It is relatively conserved among all the desaturases in the alignment except for 6803_DesA and AtFAD6 in which Leu is replaced by another hydrophobic residue, Met. However, in the model template, mSCD1 possesses a Thr at this position. Therefore, we chose to test the effects of mutations at position 287 to Thr. As shown in Fig. 4*A*, L287T indeed enables Fm1 to produce fewer ALA relative to native Fm1, further confirming the importance of the residues in the same helix.

### Mutagenesis based on comparison of Fm1 and Fm2

*F. verticillioides* 7600 has two bifunctional 12/ω3-desaturases, *i.e.* Fm1 and Fm2. Fm1 preferentially produces more ω3 products, whereas Fm2 is mainly an LA producer. We added Fm2 to the sequence alignment (Fig. S1) and compared the sequences of these two enzymes to identify residues that could discriminate their product specificity. We focused on sites where neither Fm1 and Fm2 nor Δ12 and ω3 groups share identity. This led to 14 candidates at locations 25, 67, 121, 153, 164, 190, 194, 207, 210, 240, 264, 271, 345, and 396. These sites in Fm1 were mutated to corresponding residues in Fm2 to investigate their potential effects on specificity and activity. Interestingly, two mutations, L153R and H194Q, increased the overall activity of Fm1 by 106 and 90%, respectively (Fig. 4*B*). In addition, H194Q shifted the Fm1 product profile to the preferential production of LA with an ALA/LA ratio of ∼0.5. When we mapped these two sites in the Fm1 model, we found that, like Phe^157^, Leu^153^ is very close to the catalytic iron ions. Therefore, it might also participate in fine-tuning active-site geometry, thereby increasing Fm1 activity. His^194^ is in a small helix adjacent to Phe^157^, which is very close to the upper edge of the substrate-binding pocket, suggesting that it might influence the orientation of the secondary structural elements, thereby affecting Fm1 activity.

### Stacking beneficial mutations to enhance Fm1 activity

**Figure 5.**
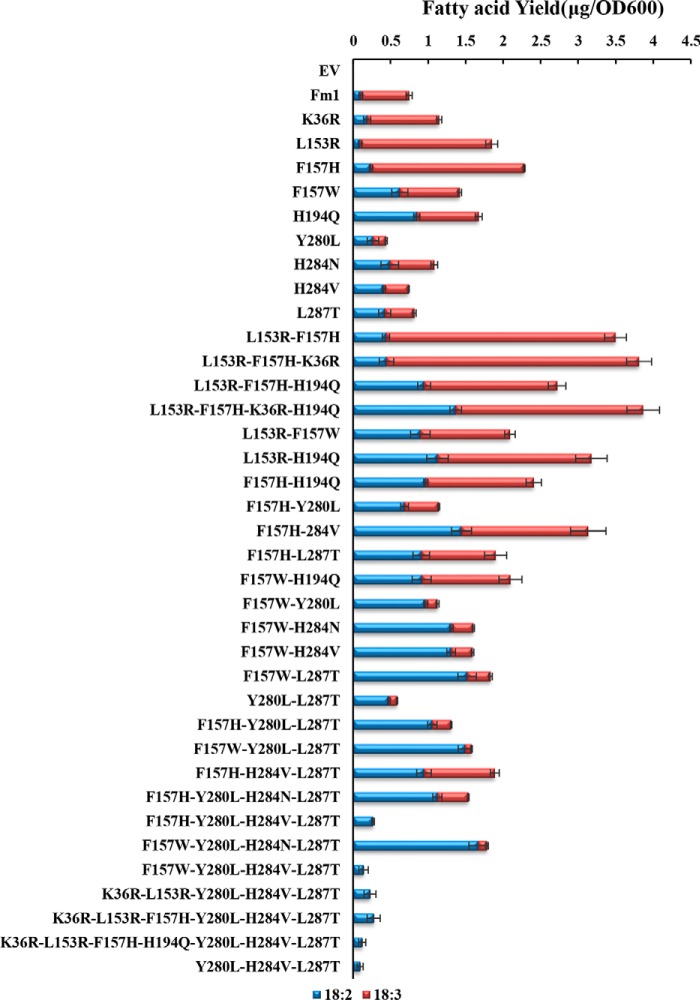
**Activity analyses for multiple site mutants.** The beneficial mutations are combined and analyzed for their activities. *Error bars* show the S.D. from three independent replicates. *EV*, empty vector.

The above analyses reveal a series of residues that affect Fm1 overall activity and product specificity: mutations K36R, L153R, and F157H increase the activity of Fm1 by more than 25%; Y280L, H284V, and L287T reverse the product specificity (*i.e.* from preferential production of ALA to LA); and F157W, H194Q, and H284N have effects on both activity and specificity. It is noteworthy that the increased-activity mutants are not the result of increased accumulation of the enzymes because the mutants' expression levels are similar to those of the native enzyme (Fig. S2). We next combined beneficial mutations to test whether their effects would be additive. As shown in Fig. 5, most of the activity-increasing mutations showed some additive effects. For example, K36R/L153R/F157H increased Fm1 activity by almost 3-fold with the majority of the products being ALA, consistent with each of them individually contributing to Fm1 activity. The Y280L/H284V/L287T mutant produces exclusively LA but with greatly compromised enzyme activity. Further stacking F157H or F157W in combination with Y280L/H284V/L287T partially restored Fm1 activity without altering the product profile. In contrast, F157W/Y280L/L287T produced ALA/LA at a ratio of ∼0.06, *i.e.* only about 115 that of Fm1, whereas its overall activity increased 80%. H194Q seems to have different effects in combination with other mutations. F157W/H194Q displayed a slight increase in overall activity relative to H194Q; however, the combination of these two sites did not result in a further decrease in the ALA/LA ratio. Interestingly, L153R/F157H/H194Q has impaired overall activity relative to L153R/F157H, suggesting that H194Q and F157H either have overlapping effects on enzyme activity or function negatively when present in combination. Although we were able to identify many mutants with altered activity or specificity with respect to 15-desaturation, no mutants were identified that could desaturate at the 15- but not the 12-position.

### Fm1 is likely a v + 3 desaturase that can perform processive desaturation reactions from existing double bonds

**Figure 6.**
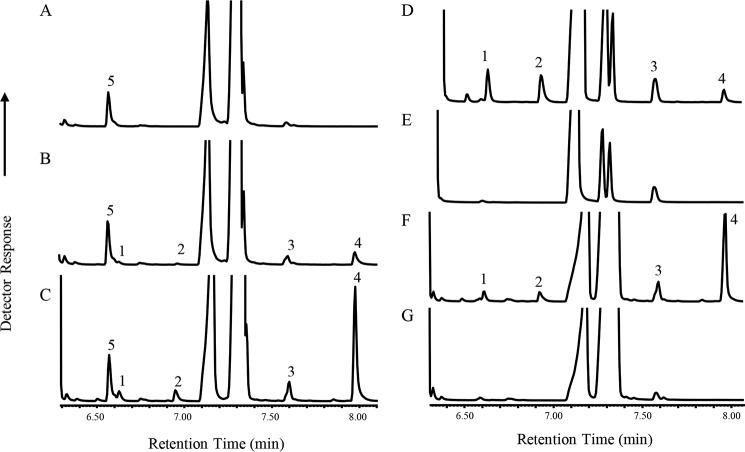
**Fatty acid profile of Fm1-expressing yeast strains.** *A*, empty vector; *B*, Fm1; *C*, Fm1-3; *D*, Fm1-3 with 16:1Δ9 feeding; *E*, empty vector with 16:1Δ9 feeding; *F*, Fm1-3 without 16:1Δ9 feeding; *G*, empty vector without 16:1Δ9 feeding. *1*, 16:2; *2*, 16:3; *3*, 18:2; *4*, 18:3; *5*, internal standard 17:0.

Fm1 can insert a double bond at the 15(ω3)-position in LA to produce ALA. Two possible mechanisms could account for this activity. In the first scenario, the enzyme could perform an ω3-desaturation, counting three carbons from the methyl end of linoleic acid in a manner like that of FAD3. A second possibility could be that Fm1 performs a desaturation by counting three carbons from the existing Δ12 double bond (*v* + 3 desaturation). To distinguish between these two possibilities, we examined the products of Fm1 with respect to 16-carbon substrate. Fm1 produces 16:2 and 16:3 from 16:1Δ9 but with much lower activity relative to that with 18:1Δ9 (Fig. 6*B*), which precluded reliable double bond positional analysis. We therefore took advantage of the enhanced-activity mutant Fm1-K36R/L153R/F157H (abbreviated as Fm1-3 hereafter), which is more active but shows the same product profile as native Fm1 (Fig. 6, *A* and *B*). When we fed the Fm1-3-expressing strain with palmitoleic acid, the production of both 16:2 and 16:3 was enhanced (Fig. 6, *D* and *F*). Double bond positional analysis from pyrrolidide derivatives identified the desaturation products as 16:2Δ9,12 and 16:3Δ9,12,15, respectively (Fig. S4). These results indicated that Fm1 processively desaturates its 16:1Δ9 substrate to 16:2Δ9,12 and 16:3Δ9,12,15 via two sequential *v* + 3 desaturation reactions.

## Discussion

Direct structure–function analysis of Fm1 is hampered by the lack of an available crystal structure. However, there are several structures of distantly related desaturases from mouse and human. Although the homology between the mammalian desaturases and Fm1 is low, the presence of a highly conserved tripartite motif was sufficient to create a plausible model of Fm1 (26). We used this structural model in conjunction with sequence comparison to identify candidate specificity-determining residues and tested them using site-directed mutagenesis. Our approach identified two spatially distinct clusters: cluster I, consisting of residues Leu^153^, Phe^157^, and His^194^ located close to the catalytic iron ions, that primarily affects catalytic activity and cluster II, comprising residues Tyr^280^, His^284^, and Leu^287^ on consecutive turns of a helix that forms the entrance of the substrate-binding pocket, that is involved in specifying 15(ω3)-desaturation.

Cluster I residues, when mutated to their equivalents in Fm2/mSCD1, showed substantial increase in overall desaturase activity compared with native Fm1. Phe^157^ seems to be the core residue of cluster I and is the closest to the di-iron center, whereas the other residues likely exert their influence primarily via the interaction with Phe^157^. Mutations of Phe^157^ to His or Trp led to an increase in Fm1 activity. In contrast, mutations of Phe^157^ to charged residues, *e.g.* Asp, Lys, and Arg, resulted in total loss of enzyme activity. In addition, an aliphatic residue (Leu) and amide-containing residues (Asn and Gln) at this position dramatically reduced enzyme activity, whereas Tyr, a structural analog of Phe, retains enzyme activity to some extent (Fig. S2). Phe^157^ is close to the catalytic iron ions and forms part of the substrate-binding pocket, specifically the bend in the channel that accommodates the curved acyl chain and potentially specifies the regioselective and *cis*-specific desaturation, consistent with the different product profiles of the F157H and F157W mutants.

**Figure 7.**
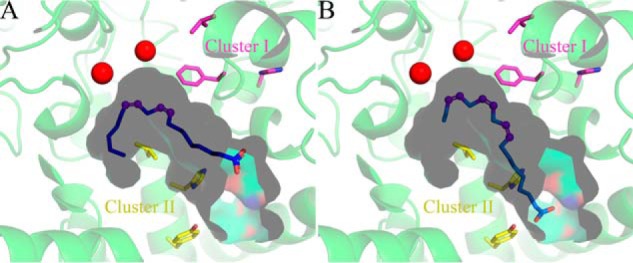
**A working model for Fm1 substrate specificity.** Mutational evidence suggests that when producing LA the headgroups of LA interact weakly with cluster II residues (*A*). In contrast, cluster II residues are critical for the production of ALA, likely because of the increased curvature of the LA substrate and its partial egress by 3-methylene groups relative to oleic acid binding (*B*).

Fm1 was useful for identifying cluster II residues, *i.e.* those contributing to 15-desaturation, because its bifunctionality allowed us to distinguish between mutations that specifically affected 15-desaturation by focusing on mutants that were still competent to perform 12-desaturation. Native Fm1 is active on 18:1 but shows only trace activity with respect to 16:1 substrate; however, the identification of cluster I residues that increased activity with C16 substrate allowed us to determine that Fm1 performs two *v* + 3 desaturations instead of a *v* + 3 followed by an ω3-desaturation. We can speculate as to how Fm1 is able to perform sequential *v* + 3 reactions (see model in Fig. 7), which require a reference interaction between a presently undefined side chain, possibly Phe^157^, and the existing double bond at C9 to position C12 adjacent to the di-iron center for desaturation. A subsequent desaturation to introduce the 15-double bond requires partial egress of the acyl chain and rebinding of the substrate such that the double bond at C12 is repositioned at the same relative orientation with respect to the di-iron center as the C9 carbon was for the first *v* + 3 desaturation. Although the headgroup specificity of Fm1 is presently unknown, the binding of acyl chain in the active-site channel would place the headgroup adjacent to the entrance of the substrate-binding pocket consisting of cluster II residues. Therefore, this shift in register is likely driven by the introduction of the *cis*-12-double bond, which creates a rigid kink in the acyl chain, that leads to strengthening of the interaction between its headgroup and cluster II residues. Our results suggest that there is some overlap between headgroup binding for the first and second *v* + 3 desaturation reactions because combinatorial mutations of cluster II residues also impaired the ability of Fm1 to perform 12-desaturation (Fig. 5). That no variants were identified in which 15-desaturation occurred in the absence of 12-desaturation is consistent with the proposed 12- followed by 15-sequential desaturation model.

Several other studies have reported efforts to define specificity determinants of 12- and 15-desaturases (23, 27). For example, a domain-swapping strategy was reported for a bifunctional oleoyl-Δ12/linoleoyl-ω3-desaturase from *A. nidulans* in which fragments of a monofunctional oleoyl-Δ12-desaturase were substituted with corresponding sections of the related bifunctional enzyme (23). Two contiguous domains that when individually substituted into the monofunctional enzyme conferred the ability to perform linoleoyl-ω3-desaturation were identified. These domains, equivalent to residues 128–145 and 146–193 of Fm1, contain two of the three residues, *i.e.* at locations 153 and 157, that affect activity, but the region corresponding to residues 280–287 that specifies linoleoyl desaturation in Fm1 was not reported for the *Aspergillus* desaturase, suggesting that they perhaps have different mechanisms. Fm1's bifunctionality is similar to a bifunctional Δ12/Δ15-desaturase from *Acanthamoeba castellanii* (AcD12) that can perform Δ12- and Δ15-desaturation processively on palmitoleic acid and oleic acid to give rise to hexadecatrienoic acid (16:3Δ9,12,15) and ALA (18:3Δ9,12,15), respectively (28). However, AcD12 can only desaturate Δ9 fatty acids, whereas Fm1 can desaturate dihomo-γ-linolenic acid (20:3Δ8,11,14), consistent with *v* + 3 activity (2). One of the resulting products, 16:3Δ9,12,15, is documented to be the precursor for synthesizing the phytotoxin sorgoleone (29). The other product, ALA, is involved in the biosynthesis of VLC-PUFAs. The existence of this bifunctional enzyme in *F. verticillioides* 7600 alleviates the necessity for a separate ω3-desaturase to synthesize PUFAs.

The evolution of enzymes capable of converting oleic acid into linolenic acid by two processive *v* + 3 desaturations in fungi and protozoans facilitates the efficient conversion of oleate to linolenate without the release of a linoleate intermediate. In higher plants, oleate is converted to linolenate via the successive action of two enzymes, FAD2 (a *v* + 3 desaturase) and FAD3 (an ω3-desaturase). We note that plants also evolved a mechanism to directly convert oleate to linolenate in which FAD2 and FAD3 monomers associate to form FAD2–FAD3 heterodimers capable of channeling the linoleate product of FAD2 directly to FAD3 (30).

In summary, we investigated the mechanism of bifunctional Δ12/ω3-desaturase Fm1. By combining sequence- and structure-based analyses with mutagenesis experiments, we have identified two clusters of residues that can affect Fm1 function. The overall activity is mainly influenced by cluster I residues, which are located in the vicinity of catalytic iron ions, whereas cluster II residues are located on the substrate-binding pocket–forming helix and primarily specify 15-desaturation activity with respect to LA. We exploited cluster I mutants to show that they desaturate 16:1Δ9 to 16:3Δ9,12,15, demonstrating that Fm1 is capable of performing sequential *v* + 3 desaturations. In addition, we have combined beneficial mutations to create mutant enzymes with significantly improved activities and altered product profiles that could potentially be useful for the production of PUFAs.

## Experimental procedures

The Δ12-, ω3-, and bifunctional membrane desaturase sequences were extracted from NCBI with the following accession numbers: AtFAD2 (AEE75153.1), 6803_DesA (WP_010872792.1), AtFAD6 (NP_194824.1), Fm1 (XP_018759876.1), Fm2 (ABB88515.1), AtFAD8 (NP_196177.1), AtFAD7 (NP_187727.1), AtFAD3 (NP_180559.1), and 6803_DesB (WP_010872924.1). Multiple sequence alignment was performed using the PROMALS3D (31) online server. The alignment results were analyzed by the Conserved Property Difference Locator (CPDL) (32) online service with manual inspections.

The *Fm1* gene was codon-optimized using JCat (http://www.jcat.de/)^3^ (34) and synthesized by GenScript (Piscataway, NJ). The synthesized gene was cloned into pCR8/GW-TOPO to generate donor vector pCR8-Fm1. To simplify protein expression, the promoter sequence of *Saccharomyces cerevisiae* translational elongation factor 1 (TEF1) (425 bp upstream of the start codon) was used to replace the original GAL1 promoter in pYES-DEST52, resulting in pTEF-DEST. TEF1 promoter is a strong constitutive promoter that allows protein expression in yeast without induction. The Fm1 yeast expression vector pTEF-Fm1 was then constructed by LR reaction of pCR8-Fm1 and pTEF-DEST.

The oligonucleotides listed in Table S1 were used to introduce point mutations into *Fm1*. Site-directed mutagenesis was performed by the QuikChange^TM^ method (Agilent Technologies). All mutants were confirmed by DNA sequencing.

The Fm1 expression vectors were introduced into *S. cerevisiae* YPH499 using Yeastmaker^TM^ Yeast Transformation System 2 (Clontech) following the manufacturer's protocols. Transformants were selected on SD-Ura plates. Positive colonies were selected and inoculated in fresh SD-Ura medium for propagation at 30 °C with constant shaking at 250 rpm. The cells were then diluted into 4 ml of fresh SD-Ura medium to a final *A*_600_ of ∼0.05 and continued to shake for 60 h at 20 °C. Cells were harvested by centrifugation at 3000 × g for 5 min and washed twice with 5 ml of double distilled H_2_O. 5 μg of heptadecanoic acid was added into each sample to serve as an internal control. The samples were dried under a nitrogen stream. Total lipid fatty acid methyl esters (FAMEs) were prepared by adding 1 ml of BCl_3_-MeOH (Sigma-Aldrich) and incubating at 100 °C for 90 min. 1 ml of double distilled H_2_O and 3 ml of hexane were added to the FAMEs and centrifuged after vortexing. The hexane phase was transferred to a new tube and dried under a nitrogen stream. The FAME products were then dissolved in 200 μl of hexane and analyzed by an Agilent GC (Model 7890A) equipped with a 5975C inert mass-selective detector with Triple-Axis detector. The samples were separated on an Agilent J&W DB-23 capillary column (30 m × 0.25 μm × 0.25 μm). The oven temperature was set to ramp up from 100 to 240 °C at a rate of 12.5 °C/min and then to 250 °C at a rate of 20 °C/min and held for 5 min. The flow rate of helium carrier gas was set at 1 ml/min. For fatty acid pyrrolidide analyses, we followed the protocol in Christie and Han (33).

For Western blot analysis, the yeast cells were collected and resuspended in a buffer containing 50 mm Tris-Cl, pH 7.5, 150 mm NaCl, 1× protease inhibitor mixture (P8215, Sigma-Aldrich), and 10% glycerol. Then the cells were disrupted by a Mini Beadbeater-8 (Biospec Products) with 0.5-mm glass beads. The homogenates were subjected to SDS-PAGE separation, transferred to a polyvinylidene difluoride membrane, and probed with a mouse anti-His_6_ antibody (GenScript).

The structure of mouse stearoyl-CoA desaturase mSCD1 (Protein Data Bank (PDB) code 4YMK) was used to model Fm1 structure by Modeler (version 9.15). We used the sequence alignment results from PROMALS3D as the input.

## Author contributions

Y. C., Q. L., and J. S. conceptualization; Y. C., X.-H. Y., and J. S. formal analysis; Y. C. and Q. L. investigation; Y. C., X.-H. Y., Q. L., C.-J. L., and J. S. methodology; Y. C. writing-original draft; X.-H. Y., C.-J. L., and J. S. writing-review and editing; Q. L. visualization; J. S. resources; J. S. supervision; J. S. funding acquisition.

## Supplementary Material

Supporting Information

## Footnotes

3 Please note that the JBC is not responsible for the long-term archiving and maintenance of this site or any other third party-hosted site.

## Footnotes

2 The abbreviations used are:

PUFA: polyunsaturated fatty acid
VLC: very long chain
LA: linoleic acid(s)
ALA: α-linolenic acid(s)
TEF1: translational elongation factor 1
SD: synthetic defined
FAME: fatty acid methyl ester.
