# Supplementary material for: Two clusters of residues contribute to the activity and substrate specificity of Fm1, a bifunctional oleate and linoleate desaturase of fungal origin
Source: J Biol Chem. 2018 Oct 22;293(51):19844–53. doi: 10.1074/jbc.RA118.005972 (PMC6314118; doi:10.1074/jbc.RA118.005972)
Supplement: Supporting Information [file supp_293_51_19844__index.html]

Two clusters of residues contribute to the activity and substrate specificity of Fm1, a bifunctional oleate and linoleate desaturase of fungal origin — Two clusters contribute to Fm1 activity and specificity — Two clusters of residues contribute to the activity and substrate specificity of Fm1, a bifunctional oleate and linoleate desaturase of fungal origin — Two clusters contribute to Fm1 activity and specificity — Supporting Information 

# Two clusters of residues contribute to the activity and substrate specificity of Fm1, a bifunctional oleate and linoleate desaturase of fungal origin

## Supporting Information

- Supporting Information (to be published online) - Supplemental Fig. 1-4.
