# Supplementary material for: Two clusters of residues contribute to the activity and substrate specificity of Fm1, a bifunctional oleate and linoleate desaturase of fungal origin
Source: J Biol Chem. 2018 Oct 22;293(51):19844–53. doi: 10.1074/jbc.RA118.005972 (PMC6314118; doi:10.1074/jbc.RA118.005972)
Supplement: Supporting Information [file supp_RA118.005972_140931_1_supp_221610_pggggg.docx]

**Supporting Information**

**Figure S1.** Alignment of Δ12 and ω3 desaturation enzymes with Fm2 sequence added in. The illustrations are shown as in figure 1.

Figure S2. Western blot of the expression levels of Fm1 mutants.

Figure S3. Summary of the activities of all the single mutants.


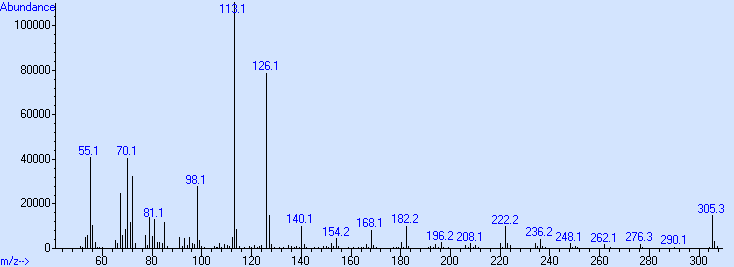

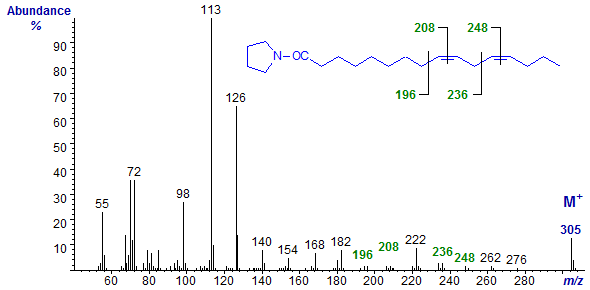


A


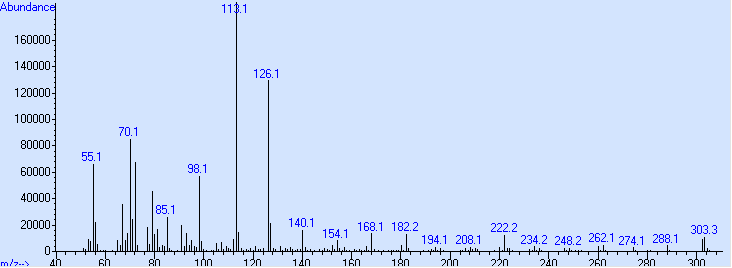


196

236


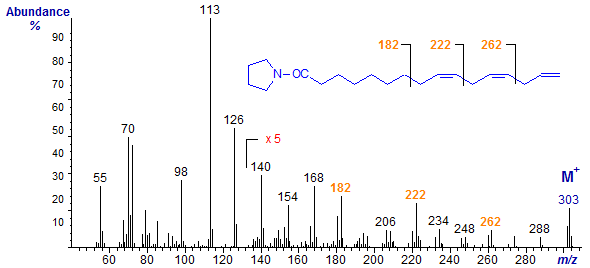


B

Figure S4. Double bond position analyses of 16 carbon fatty acids in Fm1-3 expressing yeast strain. (A) and (B) show the pyrrolidine derivatives of peak 1 (16:2Δ9,12) and 2 (16:3Δ9,12,15) in figure 6 respectively. A and B insets are the standard ion fragmentation reference for 16:2Δ9,12 and 16:3Δ9,12,15 respectively from the LipidWeb (http://www.lipidhome.co.uk/).

| **Primer** | **Sequence (5' to 3')** |  | **Primer** | **Sequence (5' to 3')** |
| --- | --- | --- | --- | --- |
| Fm1COPCR8F | aaaaagcaggctccgaattcatggctaccagacaaaga | | Fm1H194QF | aaattgatcttccagcaattgtttggttgg |
| Fm1COPCR8R | aagaaagctgggtcgaattcatctttgttccatctcat |  | Fm1H194QR | accaaacaattgctggaagatcaatttaac |
| Fm1S25FF | ttagaagcaaaatttgaaccagtattccca |  | Fm1GW198AYF | caattgtttgcgtatcaagcatatttgttt |
| Fm1S25FR | gaatactggttcaaattttgcttctaatgt |  | Fm1GW198AYR | atatgcttgatacgcaaacaattgatggaa |
| Fm1K36RF | attaagactattcgtgatgctatcccagca |  | Fm1NAS206RSPF | tatttgtttttccgttctccgtcaggtaaaggttca |
| Fm1K36RR | tgggatagcatcacgaatagtcttaatatc |  | Fm1NAS206RSPR | acctttacctgacggagaacggaaaaacaaatatgc |
| Fm1L65FF | tcagcttttgtttgggctgcattg |  | Fm1A207VF | ttgtttttcaacgtgtcttcaggtaaaggt |
| Fm1L65FR | ccaaacaaaagctgaaaccattgc |  | Fm1A207VR | tttacctgaagacacgttgaaaaacaaata |
| Fm1W67YF | tcagctttagtttatgctgcattgacttac |  | Fm1G210RF | aacgcttcttcacgtaaaggttcaaaacaa |
| Fm1W67YR | agtcaatgcagcataaactaaagctgaaac |  | Fm1G210RR | ttttgaacctttacgtgaagaagcgttgaa |
| Fm1C97GF | caaggtttgtttggcactggtgtttggatt |  | Fm1V228GF | aagtggttcagaggctcacattttgaacca |
| Fm1C97GR | ccaaacaccagtgccaaacaaaccttgaac |  | Fm1V228GR | ttcaaaatgtgagcctctgaaccacttaga |
| Fm1TG98WAF | ttgttttgttgggcggtttggattttaggt |  | Fm1S229CF | ttcagagtttgccattttgaaccaaca |
| Fm1TG98WAR | aatccaaaccgcccaacaaaacaaaccttg |  | Fm1S229CR | ttcaaaatggcaaactctgaaccactt |
| Fm1G110HF | tgtggtcatcatgcattttcattgcat |  | Fm1S229NF | tggttcagagttaaccattttgaaccaaca |
| Fm1G110HR | tgaaaatgcatgatgaccacattcatg |  | Fm1S229NR | tggttcaaaatggttaactctgaaccactt |
| Fm1N120DF | ggtaaagttaacgatgttacaggttggttc |  | Fm1SFP235AYDF | gaaccaacagcggcagtttatagagataatgaagctattttc |
| Fm1N120DR | ccaacctgtaacatcgttaactttaccatg |  | Fm1SFP235AYDR | agcttcattatctctataaactgccgctgttggttcaaaatg |
| Fm1V121IF | aaagttaacaacattacaggttggttcttg |  | Fm1V237IF | ccaacatctgcaatttttagaccaaatgaa |
| Fm1V121IR | gaaccaacctgtaatgttgttaactttacc |  | Fm1V237IR | atttggtctaaaaattgcagatgttggttc |
| Fm1V121TF | gttaacaacaccacaggttggttcttg |  | Fm1P240NF | gcagtttttagaaacaatgaagctattttc |
| Fm1V121TR | ccaacctgtggtgttgttaactttacc |  | Fm1P240NR | aatagcttcattgtttctaaaaactgcaga |
| Fm1WFL124LIFF | gttacaggtctgatttttcattcatttttgttg |  | Fm1ID248TTF | attttcattttgacctctaccattggtttggctttg |
| Fm1WFL124LIFR | aaatgaatgaaaaatcagacctgtaacgttgtt |  | Fm1ID248TTR | agccaaaccaatggtagaggtcaaaatgaaaatagc |
| Fm1L130IF | cattcatttatcttggttccatacttc |  | Fm1S264VF | ttgtacttcgctgtgaagcaagttggtgtt |
| Fm1L130IR | tggaaccaagataaatgaatgcaagaa |  | Fm1S264VR | accaacttgcttcacagcgaagtacaatgc |
| Fm1S136GF | gttccatacttcggctggaagtactcacat |  | Fm1T271NF | gttggtgtttctaacatcttgtttttatat |
| Fm1S136GR | tgagtacttccagccgaagtatggaaccaa |  | Fm1T271NR | taaaaacaagatgttagaaacaccaacttg |
| Fm1Y139IF | ttctcttggaagatttcacatcatagacat |  | Fm1L273AF | gtttctacaatcgcgtttttatatttggtt |
| Fm1Y139IR | tctatgatgtgaaatcttccaagagaagta |  | Fm1L273AR | caaatataaaaacgcgattgtagaaacacc |
| Fm1R143TF | tactcacatcatacccatcatagattcact |  | Fm1Y280LF | ttggttccactgttatgggttcatcat |
| Fm1R143TR | gaatctatgatgggtatgatgtgagtactt |  | Fm1Y280LR | aacccataacagtggaaccaaatataa |
| Fm1T148CF | catagattctgcggtcacatggatttg |  | Fm1L281IF | gttccatacatttgggttcatcattgg |
| Fm1T148CR | catgtgaccgcagaatctatgatgtct |  | Fm1L281IR | atgaacccaaatgtatggaaccaaata |
| Fm1T148DF | catagattcgatggtcacatggatttg |  | Fm1H284NF | ttatgggttaaccattggttggttgca |
| Fm1T148DR | catgtgaccatcgaatctatgatgtct |  | Fm1H284NR | caaccaatggttaacccataagtatgg |
| Fm1T148FF | catagattctttggtcacatggatttg |  | Fm1H284VF | ttatgggttgtgcattggttggttgca |
| Fm1T148FR | catgtgaccaaagaatctatgatgtct |  | Fm1H284VR | caaccaatgcacaacccataagtatgg |
| Fm1T148HF | catagattccatggtcacatggatttg |  | Fm1L287TF | catcattggaccgttgcaatcacttac |
| Fm1T148HR | catgtgacctaggaatctatgatgtct |  | Fm1L287TR | gattgcaacggtccaatgatgaaccca |
| Fm1T148NF | catagattcaacggtcacatggatttg |  | Fm1VI288DVF | catcattggttggatgcagtgacttacttacatcat |
| Fm1T148NR | catgtgaccgttgaatctatgatgtct |  | Fm1VI288DVR | atgtaagtaagtcactgcatccaaccaatgatgaac |
| Fm1T148SF | catagattcagcggtcacatggatttg |  | Fm1T298PF | catcatcatcatccggaattgccacattat |
| Fm1T148SR | catgtgaccgctgaatctatgatgtct |  | Fm1T298PR | atgtggcaattccggatgatgatgatgtaa |
| Fm1T148VF | catagattcgtgggtcacatggatttg |  | Fm1HTA302WRGF | acagaattgccatggtatcgtggcgaaggttggacatac |
| Fm1T148VR | catgtgacccacgaatctatgatgtct |  | Fm1HTA302WRGR | tgtccaaccttcgccacgataccatggcaattctgtatg |
| Fm1H150SF | ttcactggtagcatggatttggatatg |  | Fm1V318IF | ttagcaactattgatagagaatttggt |
| Fm1H150SR | caaatccatgctaccagtgaatctatg |  | Fm1V318IR | ttctctatcaatagttgctaaagcacc |
| Fm1L153RF | ggtcacatggatcgtgatatggcttttgtt |  | Fm1KF327NIF | ggttttattggtaaccatttgattcatggtatcatcgaa |
| Fm1L153RR | aaaagccatatcacgatccatgtgaccagt |  | Fm1KF327NIR | gatgataccatgaatcaaatggttaccaataaaaccaaa |
| Fm1F157LF2 | tggctctggttccaaagacagaa |  | Fm1E335GF | catggtatcatcggcaagcatgttgttcat |
| Fm1F157LR2 | ggaaccagagccatatccaaatc |  | Fm1E335GR | aacaacatgcttgccgatgataccatgaaa |
| Fm1F157HF | gatatggctcatgttccaaagacagaa |  | Fm1K345SF | catttgtttccaagcatcccattctacaaa |
| Fm1F157HR | ctttggaacatgagccatatccaaatc |  | Fm1K345SR | gtagaatgggatgcttggaaacaaatgatg |
| Fm1F157QF2 | tggctcaggttccaaagacagaa |  | Fm1K350NF | atcccattctacaacgctgatgaagcaaca |
| Fm1F157QR2 | ggaacctgagccatatccaaatc |  | Fm1K350NR | tgcttcatcagcgttgtagaatgggatctt |
| Fm1F157RF2 | tggctcgtgttccaaagacagaa |  | Fm1C367RF | ggtgaccattaccgtcatgatgatagatca |
| Fm1F157RR2 | ggaacacgagccatatccaaatc |  | Fm1C367RR | tctatcatcatgacggtaatggtcaccaat |
| Fm1A156SF | ttggatatgagctttgttccaaagaca |  | Fm1L377MF | tttttgggtcaaatgtggactatttttggt |
| Fm1A156SR | tggaacaaagctcatatccaaatccat |  | Fm1L377MR | aaaaatagtccacatttgacccaaaaatga |
| Fm1F157WF2 | tggcttgggttccaaagacagaa |  | Fm1I380SF | caattgtggactagctttggtacattgaag |
| Fm1F157WR2 | ggaacccaagccatatccaaatc |  | Fm1I380SR | caatgtaccaaagctagtccacaattgacc |
| Fm1F157YF2 | tggcttatgttccaaagacagaa |  | Fm1P391GF | gtagaacacgacggcgccagaccaggtgct |
| Fm1F157YR2 | ggaacataagccatatccaaatc |  | Fm1P391GR | acctggtctggcgccgtcgtgttctacata |
| Fm1E162KF | ccaaagacaaaaccaaagccatctaaa |  | Fm1A396GF | gccagaccaggtggcatgagatggaacaaa |
| Fm1E162KR | tggctttggttttgtctttggaacaaa |  | Fm1A396GR | gttccatctcatgccacctggtctggctgg |
| Fm1E162RF | gttccaaagacacgtccaaagccatctaaa |  | Fm1R398VF | ccaggtgctatggtgtggaacaaagatgaa |
| Fm1E162RR | agatggctttggacgtgtctttggaacaaa |  | Fm1R398VR | atctttgttccacaccatagcacctggtct |
| Fm1K164QF | aagacagaaccacagccatctaaatcattg |  | Fm1L153RF157HF | ggtcacatggatcgtgatatggctcatgtt |
| Fm1K164QR | tgatttagatggctgtggttctgtctttgg |  | Fm1L153RF157HR | atgagccatatcacgatccatgtgaccagt |
| Fm1P165QF | acagaaccaaagcagtctaaatcattgatg |  | Fm1L153RF157WF | ggtcacatggatcgtgatatggcttgggtt |
| Fm1P165QR | caatgatttagactgctttggttctgtctt |  | Fm1L153RF157WR | ccaagccatatcacgatccatgtgaccagt |
| Fm1P165YF | gaaccaaagtattctaaatcattgatg |  | Fm1H284VL287TF | ttatgggttgtgcattggaccgttgca |
| Fm1P165YR | tgatttagaatactttggttctgtctt |  | Fm1H284VY280LR | ggtccaatgcacaacccataacaatgg |
| Fm1K190MF | gcacaaatggttatgttgatcttccatcaa |  | Fm1H284NL287TF | ttatgggttaaccattggaccgttgca |
| Fm1K190MR | atggaagatcaacataaccatttgtgcagc |  | Fm1H284NY280LR | ggtccaatggttaacccataacaatgg |

Table S1. The oligonucleotides used in this study.
